# Variability of the medullary arcuate nucleus in humans

**DOI:** 10.1002/brb3.1133

**Published:** 2018-10-17

**Authors:** Beatrice Paradiso, Stefano Ferrero, Gaetano Thiene, Anna Maria Lavezzi

**Affiliations:** ^1^ ”Lino Rossi” Research Center for the Study and Prevention of Unexpected Perinatal Death and SIDS—Department of Biomedical, Surgical and Dental Sciences University of Milan Milan Italy; ^2^ Department of Cardiac, Thoracic and Vascular Sciences University of Padua Padua Italy; ^3^ Division of Pathology Fondazione IRCCS Ca' Granda, Ospedale Maggiore Policlinico Milan Italy

**Keywords:** adulthood, aplasia, arcuate nucleus, chemoreception, human brainstem, hypoplasia, medulla oblongata

## Abstract

**Introduction:**

The arcuate nucleus is a component of the ventral medullary surface involved in chemoreception and breathing control. The hypoplasia of this nucleus is a very frequent finding in victims of sudden unexplained fetal and infant death (from the last weeks of pregnancy to the first year of life). On the contrary, this developmental alteration is rarely present in age‐matched controls who died of defined causes. These observations lead to hypothesize that a well‐developed and functional arcuate nucleus is generally required to sustain life. The aim of this study was to investigate whether the arcuate nucleus maintains the same supposed function throughout life.

**Methods:**

We carried out neuropathological examinations of brainstems obtained from 25 adult subjects, 18 males and 7 females, aged between 34 and 89 years, who died from various causes.

**Results:**

For almost half of the cases (44%) microscopic examinations of serial histological sections of medulla oblongata showed a normal cytoarchitecture of the arcuate nucleus, extending along the pyramids. For the remaining 56% of cases, various degrees of hypodevelopment of this nucleus were observed, validated through the application of quantitative morphometric investigations, from decreased area, neuron number and volume, to full aplasia.

**Conclusions:**

These unexpected findings indicate that the involvement of the arcuate nucleus in chemoreception in adulthood is questionable, given the possibility of living until late age without this nucleus. This opens new perspectives for researchers on the role and function of the arcuate nucleus in humans from birth to old age.

## 1 INTRODUCTION

In humans, the arcuate nucleus (AN) is a group of neurons located in the brainstem on both sides of the midline at the ventral surface of the medulla oblongata, which extends between the caudal border of the pons and the caudal pole of the olive (Mikhail & Ahmed, 1975). This nucleus has been proposed as the homologue of the chemoreceptive areas in the ventral medullary surface (VMS) reported in experimental researches, responsible for mediating the ventilator responsiveness to carbon dioxide (CO_2_) (Bruce & Cherniack, 1987; Filiano, Choi, & Kinney, 1990; Millhorn & Eldridge, 1986; Paterson, Thompson, & Kinney, 2006; Zec, Filiano, & Kinney, 1997). In addition, functional magnetic resonance imaging performed on 11 human healthy volunteers (Gozal et al., 1994), revealed patterns of activation in the AN area during hypercapnic ventilatory test, further suggesting that the AN could be involved in respiratory regulation in humans.

The AN has been the focus of numerous studies performed on subjects who died in late fetal and infant age (from the last weeks of pregnancy to the first year of life). In particular, different groups of researchers have indicated that, in the first months of life, some infants are at risk of sudden infant death syndrome (SIDS) when their response to increased blood CO_2_ levels, particularly in arousal phase, is reduced (Biondo, Lavezzi, Tosi, Turconi, & Matturri, 2003; Filiano & Kinney, 1992; Kinney, Broadbelt, Haynes, Rognum, & Paterson, 2011; Kinney, 2009; Machaalani & Waters, 2014; Matturri, Biondo, Mercurio, & Rossi, 2000; Matturri, Biondo, Suarez‐Mier, & Rossi, 2002; Matturri et al., 2004; Paterson, Thompson, et al., 2006; Paterson, Trachtenberg, et al., 2006; Rubens & Sarnat, 2013). In a high percentage of cases, this abnormal reaction of SIDS infants has been correlated with the hypodevelopment of the AN, as well as other brainstem nuclei abnormalities.

These data are consistent with the systematic review on the neuropathological features of SIDS reported by Paine, Jacques, and Sebire (2014). The authors analyzed 153 studies, of which only 70 (46%) met the three essential diagnostic criteria for SIDS which are as follows: (a) the death is sudden; (b) death occurs in infants between the ages of 1 week and 1 year; (c) no alternative cause of death is found after a full postmortem and clinical assessment, including investigation of the death scene. Nine studies that have been discussed in this review concern the AN, five of which performed at the “Lino Rossi” Research Center of the Milan University (Biondo et al., 2003; Lavezzi, Ottaviani, Mauri, & Matturri, 2004; Matturri et al., 2000; Matturri et al., 2002; Matturri et al., 2004). The authors conclude that it is not possible to establish, on the basis of the reports on the literature, whether or not the arcuate nucleus has an etiological role in SIDS.

Overall, our many previous researches performed on a large SIDS cohort (over 150 cases), reported the presence of AN hypoplasia, sometimes even in mild form (delayed neuronal maturation, monolateral hypoplasia, or hypoplasia confined to one‐third of its extension), in more than half of the SIDS cases (approximately 60%). On the contrary, the AN hypodevelopment was a rare finding in age‐matched control cases died of known causes. These observations led us to hypothesize that a proper development and functionality of the AN could be necessary to sustain life. Nevertheless, the casual observation of the total absence of this nucleus in the medulla oblongata of a healthy adult subject who died for a polytrauma led us to revise our hypothesis. Therefore, with the aim to determine whether this was merely an isolated and inexplicable finding and then, that a well‐developed and functional AN is essential throughout the entire life span, we decided to analyze the AN in a cohort of adults who died of various causes.

## 2 MATERIALS AND METHODS

### 2.1 Study subjects

The study was conducted on 25 subjects (including the person who prompted this study), 18 males and 7 females aged between 34 and 89 years, who died from various causes (Table 1). In‐depth analyses of their brainstems were carried out at our Research Center in accordance with the guidelines established for the anatomopathological examination of perinatal deaths, under Italian law n.31/2006 “Regulations for Diagnostic Post Mortem Investigation in Victims of SIDS and Unexpected Fetal Death” (available at: https://users.unimi.it/centrolinorossi/files/gazz_ufficiale.pdf). The guidelines for the application of this law include in particular an in‐depth examination of the brainstem to highlight possible developmental alterations of the main neuronal centers that coordinate the vital functions. Moreover, in order to study the developmental steps of the human brain nuclei, which could also take years, the neuropathological protocol foresees, for research purpose, the examination of brains of infants aged over one year and adults.

**Table 1:** Profiles of the 25 cases of the study (18 males, 7 females; age range: 34–89 years) with death diagnosis and neuropathological results

Case no.	Sex/Age (years)	Death diagnosis	Arcuate nucleus histology	Other brainstem nuclei alterations
1	M/60	Aspergillary ulcer‐necrotic pneumonitis in pulmonary transplant	Normal	/
2	M/34	Arythmic sudden cardiac death	Aplasia	/
3	F/60	Myocardial infarction	Aplasia	/
4	M/48	Polytrauma by car accident	Bilateral hypoplasia (severe)	/
5	M/45	Cardiac transplant rejection	Bilateral hypoplasia	/
6	M/60	Polytrauma by fall	Aplasia	/
7	M/44	Polytrauma by fall	Normal	/
8	M/71	Prostatic carcinoma	Normal	/
9	F/49	Gastric carcinona	Aplasia	Pre‐Bötzinger hyperplasia
10	M/57	Gastric carcinona	Normal	/
11	M/78	Pancreatic carcinoma	Aplasia	/
12	F/55	Colon carcinoma	Bilateral hypoplasia (moderate)	Pre‐Bötzinger hyperplasia
13	M/61	Colon carcinoma	Normal	/
14	F/78	Thyroid carcinoma	Normal	/
15	M/67	Myocardial infarction	Normal	/
16	F/89	arrhythmogenic right ventricular cardiomyopathy	Aplasia	/
17	F/58	Arrhythmogenic right ventricular cardiomyopathy	Normal	/
18	M/80	Hypertrophic cardiomyopathy	Aplasia	/
19	M/79	Dilated cardiomyopathy	Partial hypoplasia (2/3 of its extension)	raphé obscurus hyperplasia
20	M/44	Hypertensive cardiomyopathy	Partial hypoplasia (monolateral, right)	/
21	M/67	Polytrauma by car accident	Aplasia	raphé obscurus hyperplasia
22	M/41	Polytrauma by car accident	Normal	/
23	F/60	Osteosarcoma	Normal	/
24	M/88	Myocardial infarction	Partial hypoplasia (monolateral, right)	Pre‐Bötzinger hyperplasia
25	M/64	Myocardial infarction	Normal	/

### 2.2 Protocol for the brainstem examination

After fixation in 10% phosphate‐buffered formalin, the brainstems were processed and embedded in paraffin from the lower third of the midbrain to the caudal pole of the olive. Two main specimens were obtained. The first specimen included the medulla oblongata, isolated through an uppercut at the caudal border of the pons and a lower cut at the beginning of the spinal cord; the second specimen included the pons and the lower third portion of the midbrain.

Transverse serial sections of the two samples were made at intervals of 50–60 µm. For each level, serial 5 µm sections were obtained, two of which were routinely stained for histological examination using hematoxylin and eosin and Klüver‐Barrera. The remaining sections were stained as deemed necessary and saved for further investigations.

The histological evaluation of the brainstem nuclei, according to the guidelines established by the Italian law, is focused on identifying the hypoglossus, the dorsal motor vagal, the tractus solitarius, the ambiguous, the pre‐Bötzinger, the inferior olivary, the raphé nuclei (obscurus and pallidus), the trigeminal, and above all the arcuate nucleus, the focus of this study, in the medulla oblongata; on the locus coeruleus, the median raphé, and the Kölliker‐Fuse nucleus in the rostral pons/caudal mesencephalon; and on the retrotrapezoid nucleus, the superior olivary complex, the magnus raphé nucleus, and the facial/parafacial complex in the caudal pons.

### 2.3 Specific protocol for the examination of the arcuate nucleus

A first examination was performed under a light microscope at different magnifications (from 0.5× to 100×) on serial histological sections obtained from the medulla oblongata, with the plates from VIII to XVIII of the classic Atlas of Olszewski and Baxter (1982) and the sections from E to J of the online Atlas of the Brainstem of Swenson (http://scicrunch.org/resolver/SCR_005967) used as references for the AN analysis.

Every time that the morphological examination of the AN largely corresponded to that represented in the Atlases, the nucleus was classified as “normal.” A diagnosis of “hypoplasia” was formulated when the AN showed decreased area in serial histological sections compared to the corresponding images of the Atlases at the same levels. In case of the total absence of the AN, in all levels, the diagnosis was of “aplasia.”

The examination of slides was performed blinded, with no prior knowledge of the cause of death. Comparison of diagnoses achieved by every pathologist was obtained employing the K Index (KI) to evaluate the inter‐observer reproducibility. A specific system for the K interpretation was used, where 0 to 0.2 is slight agreement, 0.21 to 0.40 indicates fair agreement, 0.41 to 0.60 moderate agreement, 0.61 to 0.80 strong or substantial agreement, and 0.81 to 1.00 indicates very strong or almost perfect agreement (a value of 1.0 being perfect agreement) (Landis & Koch, 1977). The application of this method in the present study produced a satisfactory KI (0.85).

In order to verify the reliability of the microscopic examinations, the cases were submitted to quantitative morphometric investigations.

### 2.4 Morphometric analysis of the AN

A quantitative method was applied on the serial sections obtained from the medulla oblongata, stained with Klüver‐Barrera. The basic investigation was carried out with an Advanced Image Data Analyzer (http://scicrunch.org/resolver/SCR_014440) on both sides of the AN. The following parameters were evaluated: (a) AN transverse area (expressed in mm^2^), (b) AN neuronal density (number of neurons with clearly defined edges and with a distinct nucleolus per mm^2^), and (c) AN volume (in mm^3^). The transverse area was calculated for every case as mean value of the measurements of the AN area, after having drawn its boundaries, on three slides: a histological section at high level of the medulla oblongata (corresponding to plate XIV and section I, respectively, of the aforementioned Atlases), a section at the median level (plate XII and section H) and a caudal section (plate X and section G). The evaluation of the neuronal density was performed in the same selected histological sections with the same criterion, that is, as mean value of the three measurements. A computer program developed by Voxblast (http://scicrunch.org/resolver/SCR_016491) was employing to obtain the three‐dimensional reconstruction (3‐D) of the whole structure, from the caudal pole of the olives to the pontomedullary junction, by using all the serial sections and in which the outer boundaries have been traced. The tracings were digitized by computer and then registered to reestablish their original positions relative to one another. The fourth ventricle was used as landmark for registration. Statistical calculations were carried out with the SPSS statistical software (http://scicrunch.org/resolver/SCR_002865). All the morphometric results were expressed as mean values and standard deviation (m ± *SD*). The statistical significance of direct comparisons between groups with normal, and defective AN was determined using the analysis of variance (*F*‐test). The selected threshold level for statistical significance was *p < *0.05.

### 2.5 Ethics statement

Permission from the Ethics Committee was not required for this study as our Research Center (The Lino Rossi Research Center at Milan University) is the national referral center for neuropathological studies under the Italian Law n. 31/2006. This study was part of a legal obligation, and therefore, the consent of the subjects involved was not required. The study was approved by the institutional review board of Milan University and by the Registry of Cardio‐Cerebro‐Vascular Pathology of the Veneto Region, Italy.

## 3 RESULTS

### 3.1 Examination of the arcuate nucleus

**Figure 1:**
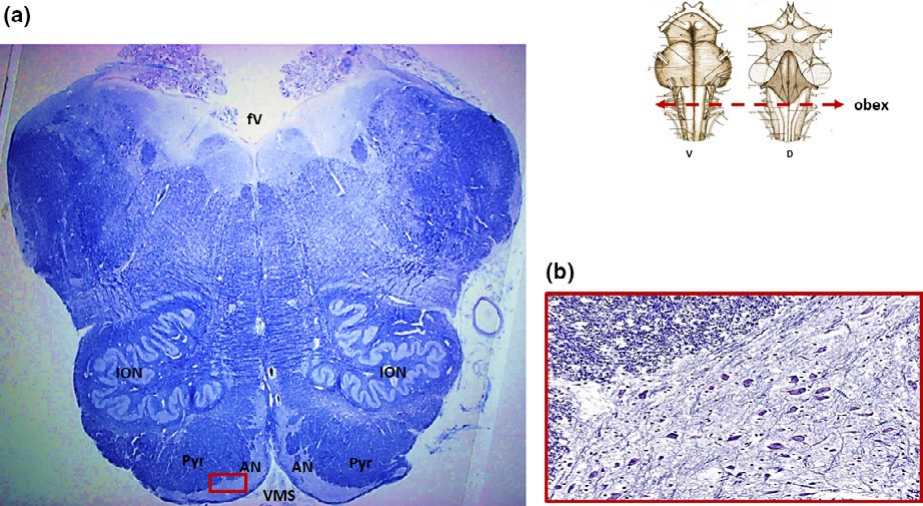
(a) Klüver‐Barrera histological transverse section of medulla oblongata at the obex level showing a normal arcuate nucleus (case n.7 of Table 1; male, 44‐year‐old). A large, well‐delineated arcuate nucleus, subdivided into two medial and lateroventral parts, is well visible adherent to the ventral medullary surface. In the framed area in (a), represented at higher magnification in (b), scattered medium‐sized, polygonal or elongated neurons are visible. At the top, schematic representation of the brainstem indicating the level of the histological section. AN: arcuate nucleus; D: dorsal; fV: fourth ventricle; ION: inferior olivary nucleus; Pyr: pyramid; V: ventral; VMS: ventral medullary surface. Magnification (a) 0.5×; (b) 20×

Histological examinations of medullary serial sections highlighted in 11 cases (44%; see Table 1) a clear, large and well‐delineated AN, subdivided in two units, extending along the ventral median fissure, superficial to the pyramidal tract, between the caudal border of the pons and the caudal pole of the olives. The extreme caudal part of the lateral extension of the AN was either triangle‐shaped or formed a narrow band. It was largest in the rostral medulla oblongata where it frequently appeared histologically continuous with the caudal pontine nuclei. The cytoarchitecture of the AN was clearly visible at the obex level (Figure 1a). At higher magnification, its neurons appeared loosely arranged, medium‐sized, oval, polygonal, or elongated with eccentric large nuclei and long dendrites frequently well discernible (Figure 1b). Occasionally, small groups of neurons, isolated or connected with the main structure, appear to be embedded among the fascicles of the pyramids. The death diagnoses of these cases were as follows: carcinoma (five cases), accidental polytrauma (two cases), myocardial infarction (two cases), aspergillus pneumonia following lung transplantation (one case), and arrhythmogenic right ventricular cardiomyopathy (one case).

**Figure 2:**
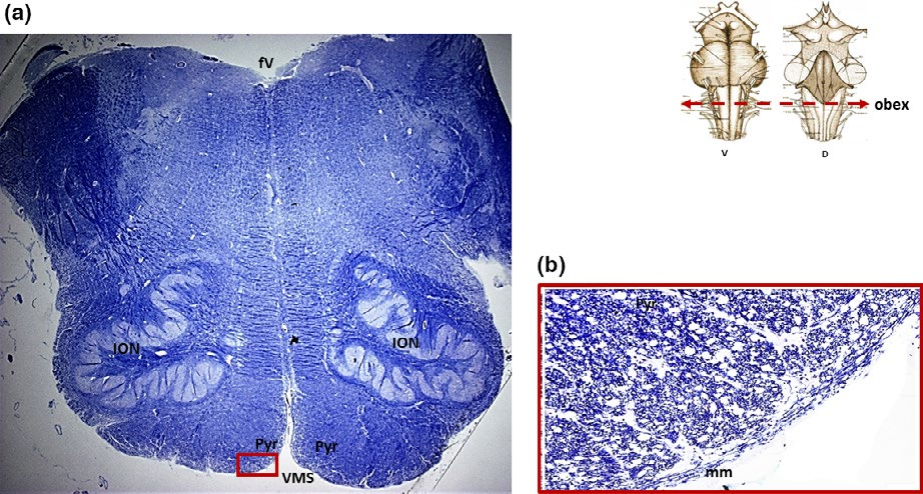
(a) Klüver‐Barrera histological transverse section of medulla oblongata showing bilateral aplasia of the arcuate nucleus (case n.11 of Table 1; male, 78‐year‐old). The framed area in (a), visible at higher magnification in (b), shows the pyramids, covered only by the meningeal pial membrane, bordering directly on the ventral medullary surface. The arcuate nucleus is completely missing. The white holes, well visible in (b) are the spaces that separate the pyramidal fascicles. D: dorsal; fV: fourth ventricle; ION: inferior olivary nucleus; mm: meningeal membrane; Pyr: pyramid; V: ventral; VMS: ventral medullary surface. Magnification (a) 0.5×; (b) 20×

For the remaining 14 cases (56%), whose deaths were not caused by respiratory diseases but due to cardiac pathologies (eight cases), carcinoma (three cases), and polytrauma (three cases), various degrees of AN hypodevelopment were highlighted at the histological examination. Precisely a “bilateral hypoplasia,” characterized by small size of the medial and lateroventral sides of the nucleus and neuronal depletion, compared with the AN features observed in the previous group, was diagnosed in three cases; a “partial hypoplasia,” including both hypoplasia of the caudal two thirds of its extension and monolateral (right) hypoplasia in three cases, and an “aplasia,” that is, the total absence of the nucleus, in eight cases (Figure 2). No difference was found between AN alterations and sex and age of the subjects. In fact, AN hypodevelopment was found in 10/18 males (56%) with a mean age of 62.3 years and in 4/7 females (57%), mean age 63.2 years.

The morphometric analysis allowed to obtain valid and objective quantitative results in support of the microscopic diagnoses. The measurements related to the AN transverse area, neuronal density and volume, allowed to subdivide the cases in three distinct categories: (I) normal (11 cases); (II) hypoplasia (with significant reduction in the AN values compared with those of the first group; 6 cases) and (III) aplasia, without any trace of the AN (eight cases).

Table 2 shows the mean values related to the three parameters (area, neuronal density, and volumetric reconstruction) obtained in Groups I and II, the only groups in which it was possible to make measurements. The mean values related to the transverse area in the first and second groups were 2.76 ± 0.5 and 1.06 ± 0.3 and the mean neuronal density 91.2 ± 4.6 and 45.3 ± 6.2, respectively. The decreased area and number of neurons observed in group II were associated to a three‐dimensional reduction of the nucleus, compared with the corresponding values obtained in group I. The mean 3‐D results were in fact 26.7 ± 6.9 in group II and 46.7 ± 1.1 in group I (*p* < 0.05). Table 3 shows the groups II and III (hypoplasia + aplasia) joined in the same category, considering both these alterations as defective expression of the AN. This enlarged group was compared to the normal one, obtaining significant statistical differences regarding the transverse area, neuronal density, and volume (*p* < 0.01).

**Table 2:** Morphometric analysis of the AN of cases with the presence of AN

Groups	*N* cases	Transverse area (mm^2^)	Neuronal density (no. cells/mm^2^)	Volume (mm^3^)
I (Normal AN)	11	2.76 ± 0.55	91.2 ± 4.6	46.7 ± 1.15
II (AN hypoplasia)	6	1.06 ± 0.3*	45.3 ± 6.2*	26.7 ± 6.9*

*Significance related to group I: *p* < 0.05.

**Table 3:** Morphometric analysis of the AN in all the 25 cases

Groups	*N* cases	Transverse area (mm^2^)	Neuronal density (no. cells/mm^2^)	Volume (mm^3^)
I (Normal AN)	11	2.76 ± 0.55	91.2 ± 4.6	46.7 ± 1.15
II Enlarged (AN hypoplasia + AN aplasia)	14	0.45 ± 0.3*	19.42 ± 14.7*	11.45 ± 9.1*

*Significance related to group I: *p* < 0.01.

### 3.2 Examination of the other brainstem nuclei

**Figure 3:**
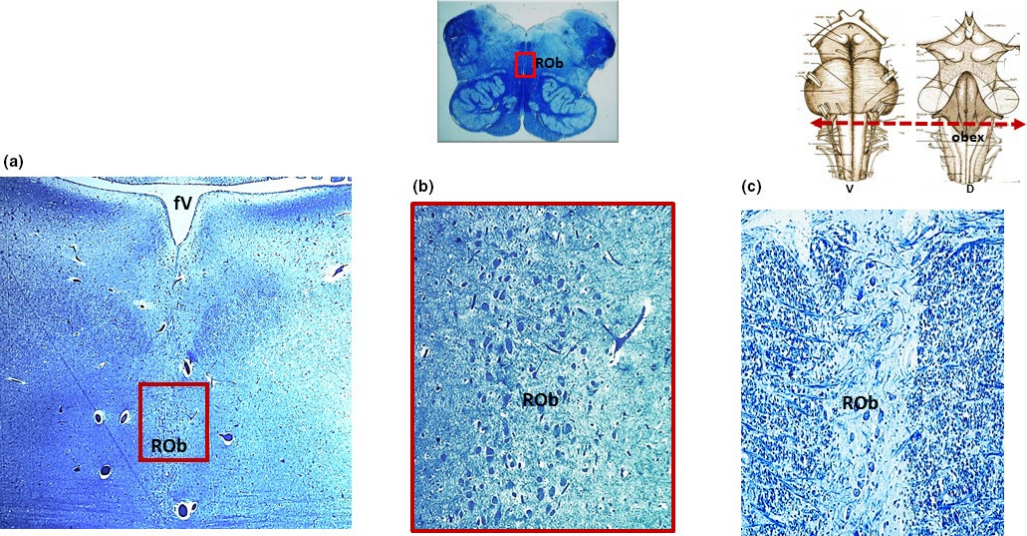
(a) Klüver‐Barrera histological transverse section of medulla oblongata showing hyperplasia of the raphé obscurus nucleus (case n.21 of Table 1; male, 67‐year‐old, with arcuate nucleus aplasia). The framed area, represented at higher magnification in (b), shows an increase in number of neurons compared with that of the raphé obscurus nucleus represented in (c) of an age‐matched case of the study (case n.22 of Table 1 with normal arcuate nucleus). At the top, on the left there is a panoramic view of a complete histological medullary section with indication in the framed area of the localization of the nucleus; on the right a schematic representation of the brainstem indicating the level of the histological section. D: dorsal; fV: fourth ventricle; ROb: raphé obscurus nucleus; V: ventral. Magnification: (a) 10×; (b) and (c) 20×

**Figure 4:**
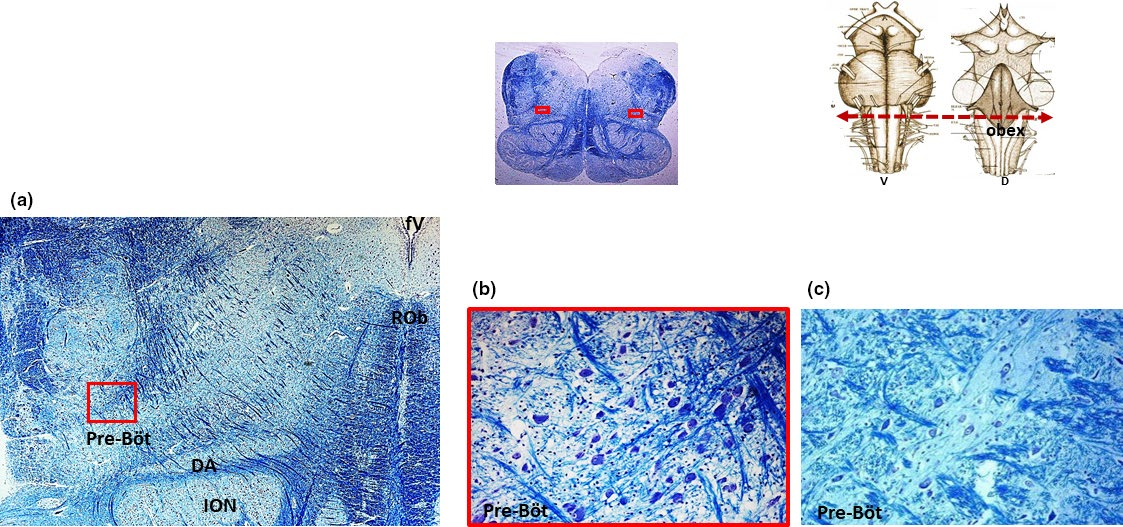
(a) Klüver‐Barrera histological transverse section of medulla oblongata showing hyperplasia of the pre‐Bötzinger nucleus, (case n.9 of Table 1; female, 49‐year‐old with arcuate nucleus aplasia). In the framed area, at higher magnification in (b), a greater number of neurons can be seen compared with that of the pre‐Bötzinger nucleus represented in (c) of another case of the study (case n.14 of Table 1 with normal arcuate nucleus). At the top, on the left there is a panoramic view of a complete histological medullary section with indication in the framed areas of the localization of the nucleus; on the right a schematic representation of the brainstem indicating the level of the histological section. D: dorsal; DA: dorsal accessory of the inferior olivary nucleus; ION: inferior olivary nucleus; pre‐Böt: pre‐Bötzinger nucleus; ROb: raphé obscurus nucleus; V: ventral. Magnification: (a) 10×; (b) and (c) 20×

It is important to note that it is sometimes difficult to identify other nuclei in the brainstems of adult humans, unlike those in fetuses and newborns for whom the neuropil is still underdeveloped, due to the presence of numerous fibers (neuronal axons, dendrites, and glial cell processes) that are synaptically intertwined and consequently conceal neurons. However, in this study it was possible to identify the locus coeruleus, the medial nucleus of the superior olivary complex, and the facial/parafacial complex in the pons; the hypoglossus, the dorsal motor vagal, the tractus solitarius, the ambiguous, the inferior olivary, and the obscurus raphé nuclei in the medulla oblongata of all the 25 cases. In two cases, one with AN hypoplasia and one with AN aplasia, a very high number of neurons in the raphé obscurus nucleus (hyperplasia) was evident as compared with the other 23 cases in which the nucleus was present with a normal cythoarchitecture, that is a moderate group of neurons arranged along the midline of the medulla oblongata (Figure 3). Moreover, the Kölliker‐Fuse nucleus and the retrotrapezoid nucleus in the pons, and the pre‐Bötzinger and pallidus raphé nucleus in the medulla oblongata were detectable in five cases, despite the presence of numerous nervous processes. Notably was the observation in two cases with AN hypoplasia and one with AN aplasia, of an overdeveloped pre‐Bötzinger nucleus (hyperplasia), characterized by a marked increased area and number of neurons compared with the same nucleus highlighted in the other two cases, in which the cytoarchitecture looked like normal, according to our previous study (Lavezzi & Matturri, 2008; Figure 4). These results are reported in Table 1.

## 4 DISCUSSION

This paper concerns the variable and surprising behavior in adult humans of the medullary AN, which has been hypothesized to have a central chemoreceptive function and discusses the possible significance of its hypoplasia in advanced age.

Respiratory activity must respond to chemosensory stimuli in order to maintain O_2_ and CO_2_ homeostasis in blood and tissues. In particular, the CNS mechanism for CO_2_ detection and ventilatory regulation, mainly deduced from experimental studies, involves the neurons located in chemosensitive areas of the VMS. Since the neurons of the human AN have similar characteristics to those of VMS, it has been hypothesized that the AN contributes to the regulation of respiration in humans. The implication of the AN in various lung dysfunctions reinforces this supposition (Folgering, Kuyper, & Kille, 1979; Matturri et al., 2003; Ono et al., 2001). However, at this moment, no proven functional information was provided about the role of the human AN in chemoreception and breathing control. Also in the study of Zec et al. (1997) performed on human fetal brainstems, the involvement of the AN in ventilation is only supposed but not demonstrated. These authors, by using a lypofilic dye (DiI) in order to examine anatomic relationships of the human AN with cardiorespiratory‐related brainstem regions, observed the presence of labeled fibers from the AN to the caudal raphé, a region implicated in respiratory control. However, because the staining density obscured the underlying cytoarchitecture, this study could not confirm that the DiI fibers originate from AN neurons.

Furthermore, Filiano et al. (1990) comparing the human and feline superficial ventral medulla, only supported the idea that the neurons of the human AN are homologous to the neurons in chemosensitive areas of the cat.

The underdevelopment of the AN, in all its forms (delayed neuronal maturation, hypoplasia, and aplasia), has been observed in over 50% of the victims of sudden perinatal death, as reported in previous studies carried out at our center (Biondo et al., 2003; Matturri et al., 2000; Matturri et al., 2002; Matturri et al., 2004) and in similar studies conducted by other groups of researches (Kinney, 2009; Kinney et al., 2011; Machaalani & Waters, 2014; Paterson, Thompson, et al., 2006; Rubens & Sarnat, 2013), thus supporting the hypothesis that this nucleus could be vitally important for the chemoreception, though however without trying a causal relationship with SIDS. We must also consider that other developmental irregularities could contribute to the pathogenetic mechanism of death.

The results obtained on the AN in adult human led us to hypothesize that the function of any brainstem nucleus may not be constant and equally important throughout life but it can markedly change with age. We wish to suggest that, while some nuclei could give an essential contribution through the entire life span, others, like the AN, only during the earliest developmental stages. This is perhaps just our likely hypothesis, based on our previous research on the intermediolateral nucleus (ILN) in the spinal cord (Lavezzi, Corna, Mehboob, & Matturri, 2010). In fact, the activity of the ILN is essential in the first phases of fetal life, as it is able to promote spontaneous bursts of rhythmic respiratory‐related activity, that are essential for the lung development, even in the absence of excitatory synaptic drive, through the release of neurotrophic signals, and modifications of the intracellular milieu (Ren & Greer, 2003; Ren, Momose‐Sato, Sato, & Gree, 2006; Spanswick & Logan, 1990). Moreover, when completely differentiated, in postnatal life, the ILN no longer plays a leading role in the respiratory pattern but it acquires a new function as effector of orthosympathetic reflexes (Powley, 2013).

Besides, we believe that the role and structure of a given nucleus may change from one subject to another in adulthood, and this applies above all to the AN, since its morphology is extremely variable in age‐matched subjects, as shown in this study. Baizer (2014), in an interesting review on the cytoarchitectonic organization of the human brainstem compared to that of several other species, specified that the AN is a unique structure in humans, not present on other species (as cat, squirrel monkey, and macaque monkey) with the exception of the chimpanzee in which it is sometimes observable. She reported, though in a limited number of cases (four females and four males aged from 45 to 71 years) provided by the Witelson Normal Brain Collection (Witelson & mcCulloch, 1991), individual variability of the AN in the size and shape and frequent asymmetry with left–right differences, without any mention of its hypodevelopment or aplasia.

Our observations raise an important question, that is, the AN anomalies reported in this study are developmental in origin or acquired? If congenital, the hypothesis of an important role of the AN in chemoreception and gas homeostasis control would be unsustainable, given the possibility of survival for a long time in the absence of this nucleus. If acquired, they could be secondary to an injury, as already suggested for SIDS infants with AN hypoplasia (Lavezzi et al., 2004). In these cases, death was attributed to a failure in homeostatic control in the presence of a stressor, such as hypoxia or increased CO_2_, which are frequently associated with tobacco smoke exposure. We can also offer another possible explanation of the presence of AN hypoplasia in adulthood, based on the triple risk model for the pathogenesis of SIDS proposed by Filiano and Kinney (1994). According to this model, SIDS results from the intersection of three overlapping factors: (1) a vulnerable infant; (2) a critical developmental period in homeostatic control, and (3) an exogenous stressor(s). Essentially, the infant’s vulnerability lies latent until he/she in the critical period is not able to compensate an exogenous stress, such as the hypercarbia due to the face down position. However, if an infant with congenital arcuate nucleus hypoplasia passes through the first year of life (according to the definition of SIDS age range) without meeting an exogenous stressor, or if he/she is able to compensate this factor with one of multiple back up chemosensory systems, can go unharmed beyond this period and show the hypoplasia in adulthood. Or again we may consider more simply that hypoplasia of the AN could be the result of a normal physiological aging process leading to neuron loss. For many years, it has been reported in the literature that extensive neuron death is an inevitable result of normal aging (Brody, 1992; Dayan, 1970a, 1970b). Morrison and Hof (1997) highlighted, however, in a wide review on the aging brain, how the application of stereological techniques allowed to demonstrate extensive loss of neurons in cerebral cortex of subjects affected by Alzheimer’s disease (AD). On the contrary, in neurologically normal elderly individuals there was no evidence of neuron number decline in the same region. More recently, Bishop, Lu, and Yankner (2010), in another review on the same topic, refer that, although neuronal loss is minimal in most cortical regions, age‐dependent changes, as reduced synaptic connectivity and loss of integrated functions, can occur in different brain regions.

Anyway, in support of the hypothesis of a diminished important role of AN in adulthood is the observation, here triggered by the morphometric investigation, of a significant decrease on neuronal density, even in a normal AN structure, compared with that of infants died in the first year of life. In fact, the mean neuronal density observed in infants with a well‐structured AN in a previous study of our Research Center (Matturri et al., 2000) was 236 ± 14, more than twice greater than that here obtained in adults (mean value: 91.2 ± 4.6).

Noteworthy is the association here showed, although in a few cases, between AN hypoplasia/aplasia and hyperplasia of two chemosensitive centers of the medulla oblongata involved in ventilator drive, the pre‐Bötzinger nucleus, and the raphé obscurus nucleus. The pre‐Bötzinger is a nucleus that contains a limited number of small neurons very essential for rhythmogenesis (Lavezzi & Matturri, 2008; Ramirez, 2011). Experimental investigations have in fact shown that this neuronal structure generates constantly the inspiratory phase of respiratory rhythm (Lü, Zhang, & Duan, 2017; Rekling & Feldman, 1998; Smith, Ellenberger, Ballanyi, Richter, & Feldman, 1991). Schwarzacher, Rüb, and Deller (2011), by using comparative cytoarchitectonic criteria, confirmed the relevance of the pre‐ Bötzinger in the neuronal control of eupneic breathing in humans. The raphé obscurus nucleus contains instead serotonergic neurons acting as CO_2_ sensors that are capable of maintaining pH homeostasis, so playing an important role in breathing control (Ozawa & Okado, 2002; Paterson, Trachtenberg, et al., 2006). An increase in the number of neurons in these structures, even if observed in a very low percentage of cases, may be interpreted as an attempt to compensate the alleged reduced chemoreceptive functionality of the AN.

A limitation of this study is the low number and heterogeneity of the investigated subjects. Therefore, we intend to continue this research on a large series of cases, with the aim to perform a more detailed analysis of the AN in homogeneous subgroups and to highlight possible sex, age, and death cause differences. There are in fact important biological differences between men and women regarding epidemiology and pathophysiology of many widespread diseases (Regitz‐Zagrosek, 2012). Furthermore, we want to evaluate the neuroanatomic connections of the AN, via interneuronal synapses, with other nerve centers with the aim to provide valuable insight on the variable behavior of the AN in human adults. Already, Edlow, McNab, Witzel, and Kinney (2016), using ultra‐high resolution diffusion spectrum imaging tractography, have recently demonstrated in healthy adults the presence of integrated central homeostatic networks (CHN) between autonomic and cardiorespiratory nuclei in the human brainstem and forebrain sites critical to homeostatic control. A “CHN connectome” has been defined in particular by these authors between the raphé nuclei in the brainstem and the medial temporal lobe. Our aim is to carry out a similar study not only to verify the links of the AN with the raphé obscurus and the pre‐Bötzinger nuclei, but also to highlight new possible connectivity patterns with higher forebrain centers relevant for the autonomic control, with the possibility to modulate one another. A reduced presence of the AN in adulthood could be counterbalanced by an increased expression and functionality of the other components of the network, to maintain a stable homeostasis. This interpretation could be an initial step toward elucidating the neuroanatomic significance of the intersubject variability of the AN nucleus in adult humans.

In conclusion, this study does not want to give an explanation about the different morphology and behavior of the AN we have observed in adults, but only to expose our observations hoping to stimulate researches on the role and function of this nucleus from birth to old age.

## CONFLICT OF INTEREST

The authors declare that they have no conflict of interest.

## AUTHOR CONTRIBUTIONS

AML, BP, GT, and SF planned and carried out the research and contributed to the analysis and interpretation of the results. All the authors drafted and approved the final version of the manuscript.
